# Mapping of the Covid-19 Vaccine Uptake Determinants From Mining Twitter Data

**DOI:** 10.1109/ACCESS.2021.3115554

**Published:** 2021-09-24

**Authors:** Anna Baj-Rogowska

**Affiliations:** Department of Informatics in ManagementGdañsk University of Technology 49646 80-233 Gdañsk Poland

**Keywords:** 6As taxonomy, Covid-19, determinants of vaccine uptake, immunization hesitancy, SARS-CoV-2, vaccination, vaccine acceptance, vaccine hesitancy

## Abstract

Opinion polls on vaccine uptake clearly show that Covid-19 vaccine hesitancy is increasing worldwide. Thus, reaching herd immunity not only depends on the efficacy of the vaccine itself, but also on overcoming this hesitancy of uptake in the population. In this study, we revealed the determinants regarding vaccination directly from people’s opinions on Twitter, based on the framework of the 6As taxonomy. Covid-19 vaccine acceptance depends mostly on the characteristics of new vaccines (i.e. their safety, side effects, effectiveness, etc.), and the national vaccination strategy (i.e. immunization schedules, quantities of vaccination points and their localization, etc.), which should focus on increasing citizens’ awareness, among various other factors. The results of this study point to areas for potentially improving mass campaigns of Covid-19 immunization to increase vaccine uptake and its coverage and also provide insight into possible directions of future research.

## I. Introduction

According to current knowledge, mass vaccination is the only way to contain the spread of the SARS-CoV-2 virus, the cause of the Covid-19 pandemic. To bring this pandemic to an end, a large proportion of the world needs to be immune to the SARS-CoV-2 virus. Herd immunity is a key concept for pandemic control and its extinction [9]. However, to achieve herd immunity and cut the transmission chain, using a vaccine with a claimed 95% efficacy, we need to vaccinate at least 63% to 76% of the population [7]. This required vaccine coverage is certainly very high, and may not be easily attained for many reasons. This is a huge challenge not only for pharmaceutical companies and finite healthcare resources, but also for government agencies and regulatory authorities [8], [9], [31].

Reference [10] highlighted the role of vaccination programmes, which must be effective and widely adopted. The observed poor uptake of vaccines in the population makes it difficult to limit the negative impact of Covid-19 on health worldwide. Statistics show that the percentage of citizens who have received at least one dose of the vaccine in the European Union (EU) is around 50% [6]. Some countries exceed this average, such as Germany - 53%, and Finland - almost 60%; however, vaccination rates are significantly off target. While, previously, the biggest problem with the vaccination program was low supply, today it is low demand. Many people do not want to be vaccinated.

Despite the fact that governments are taking a wide range of measures in response to the Covid-19 outbreak, effective ways to encourage citizens to vaccinate are hard to find. To achieve the goals of the vaccination policy, in addition to overcoming the logistical and supply challenges, it is extremely important to counteract the reluctance to vaccinate, which is steadily growing. Vaccine hesitancy is a complex issue driven by a mix of demographic, social and behavioral factors. Determinants concerning vaccine uptake are complex and context-specific, as they vary according to the time, place and severity of the disease and the vaccine characteristics [5].

Many reviews have focused on the classification of possible determinants of vaccine aversion and the wider uptake of different vaccines, for example, the uptake of the influenza vaccine by older people [3], the tetanus/diphtheria/polio vaccine for children [4] or childhood vaccines till ≤7 years of age [5]. In the face of the current Covid-19 pandemic, a pragmatic methodology (beyond questionnaire experiments) is needed to reach the main determinants of Covid-19 vaccine acceptance, which is lacking in the literature. For that reason, this study aims to fill this gap. The study is based on text data obtained from Twitter regarding vaccines in Poland. By applying (i) a taxonomy model of 5As, and (ii) a bottom-up approach during data analysis - the mining of tweets from the public discussion provided the topics, and finally, after further analysis, a set of key determinants of vaccine uptake was obtained, and the model was expanded with another dimension labeled *Assurance*, thus forming 6As.

The proposed approach (i) examines the main determinants of vaccine uptake, (ii) identifies possible root causes of non-vaccination, (iii) outlines the relevance of the determinants for citizens’ perceptions, and (iv) can support the subsequent design of robust and evidence-based interventions by governments. Reaching the main determinants of vaccine uptake can help with designing and targeting vaccination strategies, in order to gain extensive acceptance in the population. This is a key path to ensuring a fast liberation from the Covid-19 pandemic.

The main contribution of this paper is (i) the identification of an additional sixth dimension in the 5As taxonomy, labeled *Assurance*; (ii) a preliminary proof-of-concept of the 6As; (iii) a validation of the usability of textual data from public discussions in identifying and classifying the determinants of vaccine uptake; (iv) the development of a bottom-up methodology for the examined issues.

The remainder of this paper is structured as follows. First, we review the background and relevant literature in Section 2. Section 3 introduces the research methodology. Section 4 presents the empirical results obtained in the study, with a discussion of the findings and implications. Finally, Section 5 concludes the study.

## II. Theoretical Background and Related Studies

Vaccine ‘hesitancy’ is an emerging term in the scientific literature and public discourse (i.a. social media) on vaccine decision-making and the determinants of vaccine uptake. The reasons behind decisions to refuse or delay vaccination are varied and context-specific, thus there is no single form that vaccine hesitancy takes [11]. According to [2], the acceptance and adherence to public health recommendations by the population depend largely on the way people perceive a threat. The study of [12] revealed a comprehensive list of concerns related to the Covid-19 immunization of people who do not wish to be vaccinated. Respondents most frequently reported: lack of proper testing of vaccines (74.1%), vaccine adverse effects (65.1%), lack of vaccine effectiveness (44.9%) and improper transport/storage of vaccines (14%). However, the results of campaigns to encourage vaccination are not only dependent on vaccine efficacy and safety. Effective communication campaigns are needed, based on transparency and focusing on restoring trust in authorities, the government and medical professionals [14]. According to [13], vaccine acceptance among the general public and healthcare workers plays a crucial role in the successful control of the pandemic. We can consider immunization programs to be effective when there are high rates of coverage and acceptance in the population [15]. To achieve this, detecting the determinants of Covid-19 vaccine acceptance is crucial.

Reference [16] distinguished the determinants of Covid-19 vaccine acceptance, based on textual data collected from Weibo, a crucial public opinion platform in China. The main determinants of Covid-19 vaccine acceptance in China included the price and side effects. In turn, the study of [17] aimed to assess the prevalence of the acceptance of the Covid-19 vaccine, and the determinants of this among people in Saudi Arabia. By usage of a questionnaire, the researchers found perceived risk and trust in the health system to be significant predictors of the uptake of the Covid-19 vaccine.

The work of [18] focused on examining Covid-19 vaccine acceptance rates in Russia. The study identified a wide range of factors associated with Covid-19 vaccine uptake, which were grouped into the following main areas: sociodemographic and health-related characteristics, cues to action, perceived benefits and barriers. When the vaccine was proven to be safe and effective, the rate of vaccine acceptance increased. Moreover, gender and income significantly influenced the acceptance rates. Whereas [19] examined the individual, communication, and social determinants associated with vaccine uptake. Their study identified ethnicity, risk perceptions, exposure to different media for Covid-19 news, party identification, and confidence in scientists as factors that would affect Covid-19 vaccine uptake.

A review of previous research on vaccine uptake (see: Table 1) indicates that this phenomenon is increasingly gaining academic attention. Facing the fast-paced dynamic of the coronavirus pandemic, researchers use the different environments to collect data and use a variety of methods for data analysis. The rapid and easily accessible environment of social media, here namely Twitter, is popular and very often used to gain international insights into public opinion on the Covid-19 vaccination. However, a lack of research dedicated to the usage of the 5As framework is clearly visible.

**TABLE 1:** Overview of Studies With Different Approaches to Analysis

No	Authors	The main goal of the study	Country	Source of data	Methods used	Results/Conclusion	Determinants	Taxonomy/model
1	[46]	Analyzing public sentiment on the Covid-19 vaccination and the aftermath of vaccination regarding health safety measures.	US	Tweets in English, collected in April–May 2021	Natural language processing and sentiment analysis techniques	People have positive sentiments towards taking Covid-19 vaccines despite certain adverse effects of some of the vaccines. Their forecast model predicted that around 62.44% and 48% of the US population would receive at least one dose of vaccine and be fully vaccinated, respectively, by the end of July 2021.	Not applied	Not applied
2	[47]	Examining public discussions and emotions using Covid-19 related messages on Twitter.	Worldwide	Tweets only in English, collected from March 1 to April 21 in 2020	Machine learning approach, Latent Dirichlet Allocation (LDA) and sentiment analysis	Identification of popular unigrams and topics. Real-time monitoring and assessment of the Twitter discussion and concerns raised can be promising for public health emergency responses and planning.	Not applied	Not applied
3	[48]	Understanding the public’s perception of the safety and acceptance of Covid-19 vaccines in real-time by using Twitter polls.	Worldwide, English, Spanish, or other languages	Two polls by using Twitter’s built-in, anonymous polling tool	Twitter Poll Analysis	Despite the perceived high level of uncertainty regarding the safety of the available Covid-19 vaccines, the authors observed an elevated willingness to undergo vaccination among their study sample.	Not applied	Not applied
4	[49]	Identification of the topics and sentiments in the public Covid-19 vaccine-related discussion on social media.	Global perspective	Twitter chatter dataset from March 11, 2020 to January 31, 2021	Machine learning approach, Latent Dirichlet Allocation (LDA) and sentiment analysis	16 topics were obtained, which were grouped into 5 overarching themes. The topics mirrored the active news in the mainstream media. The positive sentiment around Covid-19 vaccines and the dominant emotion of trust shown in the social media discussion may imply a higher acceptance of vaccines.	Not applied	Not applied
5	[50]	The illustrating of public attitudes towards mask usage during the Covid-19 pandemic, from Twitter data.	Worldwide in the English language	Twitter data only in English, collected from March 17, 2020 to July 27, 2020	NLP, clustering and sentiment analysis techniques	Topic clustering based on mask-related Twitter data offers revealing insights into societal perceptions of Covid-19 and techniques for its prevention. The volume and polarity of mask-related tweets greatly increased.	Not applied	Not applied
6	[16]	Conducting a country-specific study of real-time public awareness and behavioral responses to Covid-19 vaccines and vaccination in China.	China	Weibo chatter data in Chinese (Simplified Chinese and Traditional Chinese) collected from January to October 2020	Natural language processing and sentiment analysis techniques	The Chinese public is divided in terms of vaccination prices and has differing expectations. Topics on Covid-19 vaccine acceptance in China include price and side effects.	Price and side effects	Not applied
7	[51]	The investigation of determinants, describing a diverse set of socio-economic characteristics, in explaining the outcome of the first wave of the coronavirus pandemic.	Worldwide	A review of the literature describing the social and economic factors which contribute to the spread of an epidemic.	The Bayesian model averaging (BMA) technique	The examination of a total of 31 potential determinants that describe a diverse ensemble of social and economic factors, including healthcare infrastructure, societal characteristics, economic performance, demographic structure, etc.	Socio-economic determinants: the level of economic development, the population size	Not applied
8	[52]	A content analysis based on the capability, opportunity, motivation–behavior (COM-B) model to characterize the determinants influencing behavioral intentions toward Covid-19 vaccines.	Worldwide	Tweets posted in English from November 1–22, 2020	A theory-based content analysis and coding of textual data	Researchers identified tweets that contained behavioral intentions regarding Covid-19 vaccines and mapped them to constructs in the adapted model. Then, nine themes were generated that influence Twitter users’ intentions to receive Covid-19 vaccines.	Misinformation or conspiracy theories about Covid-19; the positive value of vaccination to society; the mistrust of vaccines and the government	COM-B model
9	[1]	A practical taxonomy for the determinants of vaccine uptake	Worldwide	Scientific research from 1970 to 2016	Literature review	The 5As taxonomy facilitates a mutual understanding of the root causes of poor uptake.	23 possible primary determinants of vaccination coverage	5As
10	Present work	Revealing the main determinants of Covid-19 vaccine uptake from Twitter data	Poland	Tweets in Polish collected in May 2021	Text mining, analyzing and coding textual data	(i) The identification of an additional sixth dimension, labeled Assurance, in the 5As taxonomy; (ii) a preliminary proof-of-concept of the 6As; (iii) a validation of the usability of textual data from public discussions in identifying and classifying determinants of vaccine uptake.	17 determinants have been identified; they are presented in Table 4.	6As

The studies previously referred to above provide evidence that vaccine uptake may be determined by a complex mix of demographic, social and behavioral factors. To order these factors, the present study was based on the 5As taxonomy according to [1]. They identified the determinants of vaccine uptake as 5As dimensions: *Access*, *Affordability*, *Awareness*, *Acceptance* and *Activation*. Determinants extracted from a systematic literature review had been assigned to each dimension, and this approach facilitated their understanding. Their study proved that the 5As taxonomy captured all the identified determinants of vaccine uptake. Therefore, in this study we decided to use this framework in our methodology to develop a structured classification.

A sixth dimension, labeled *Assurance*, was uncovered during the empirical stage of this study. Table 2 includes a definition for each of these six dimensions. By knowing the major determinants of vaccine uptake, actions can be better tailored to effectively improve the success rate of the vaccination program.

**TABLE 2:** Factors Creating the 6As With Their Definitions

Root cause	Definition with examples
Access	The ability of individuals to be reached by, or to reach, recommended vaccines (e.g. location or convenience of access, etc.).
Affordability	The ability of individuals to afford vaccination, both in terms of financial and non-financial costs (e.g. time, etc.).
Awareness	The degree to which individuals know the need for, and availability of, recommended vaccines and their objective benefits and risks (e.g. availability of detailed information about vaccines and vaccination schedules, etc.).
Acceptance	The degree to which individuals accept, contest or refuse vaccination (e.g. severity of disease, safety and efficacy of a vaccine, social responsibility, etc.).
Activation	The degree to which individuals are nudged towards vaccine uptake (e.g. advertising, calling or sending an SMS, etc.).
Assurance	The degree to which individuals have trust and certainty with regard to being protected, supported and cared for associated with the vaccination (e.g. preliminary medical tests before vaccination, post-vaccination medical support, insurance, etc.).

## III. Methodology

This section provides the research methodology adopted in the current study. *Section* $A$ aims to presents the method of data collection. *Section* $B$ describes data analysis. *Section* $C$ explains the bottom-up approach taken in the present study.

### A. Data Collection and Preparation

The starting point in the empirical part of the study were textual data obtained from Twitter. Discussions between users on Twitter, which constitute opinions, insights and comments on vaccines, are valuable material that, after appropriate processing, will provide new knowledge. A scraping of Twitter data was conducted via QDA Miner software, using the keywords: “covid-19” OR “vaccination”^1^ OR “vaccine”^2^ OR “covid” OR “coronavirus” OR “SARS-CoV-2” OR “Johnson & Johnson” OR “Moderna” OR “Oxford / AstraZeneca” OR “Pfizer / BioNTech”, with the period between 1^st^ to 30^th^ May 2021. This query was designed to obtain a broad spectrum of data from discussions among Twitter users about vaccinations and vaccines. We collected in total 125 495 tweets only in Polish. The Polish language is so unique that it is not used outside of Poland. The assumption of focusing only on Polish tweets was aimed at: (i) selecting only one country for evaluation as a case study; (ii) providing access to discussions regarding homogeneous government regulations on vaccination; and (iii) guaranteeing the relative universality of the results for other European Union countries, given that Poland is also a member. After collecting the data, we performed the pre-processing steps. Tweets in a language other than Polish were deleted, duplicate or empty tweets were removed, and finally, we obtained a set of 105 849 tweets ready for further data analysis.^1^In Polish: “szczepienie”.^2^In Polish: “szczepionka”.

### B. Data Analysis

First, topic modeling was performed to extract the latent topics in the tweet data using the QDA Miner software. A 33-topic model was found to be optimal in terms of the average semantic coherence of the model. As a result of this phase, we obtained topics, described by top-weighted keywords. Next, an iterative process of topic labeling was performed.

Second, we employed coding to identify relevant interactions between the topics and then aggregate them into higher-order concepts (categories of determinants). The topics were coded and classified under each dimension of the As framework. For example, the tweet extract “I came for a vaccination, but it is a pity that the vaccines did not come ”^3^ was classified as evidence of the topic concerning problems with delays in Covid-19 vaccine deliveries. When there are problems with the supply of vaccines, people who want to be vaccinated generally have a problem with vaccine uptake. Therefore, this topic was included in the *Access* dimension. Finally, as a result of this phase, we obtained 17 determinants. Then, each determinant was categorized as a representative of *Access*, *Affordability*, *Awareness*, *Acceptance, Activation* or other using the definitions of each dimension according to Table 1.^3^In Polish:”Przyjechałem na szczepienie, ale szkoda że szczepionki nie przyjechały ”

### C. The Bottom-Up Approach

**FIGURE 1.**
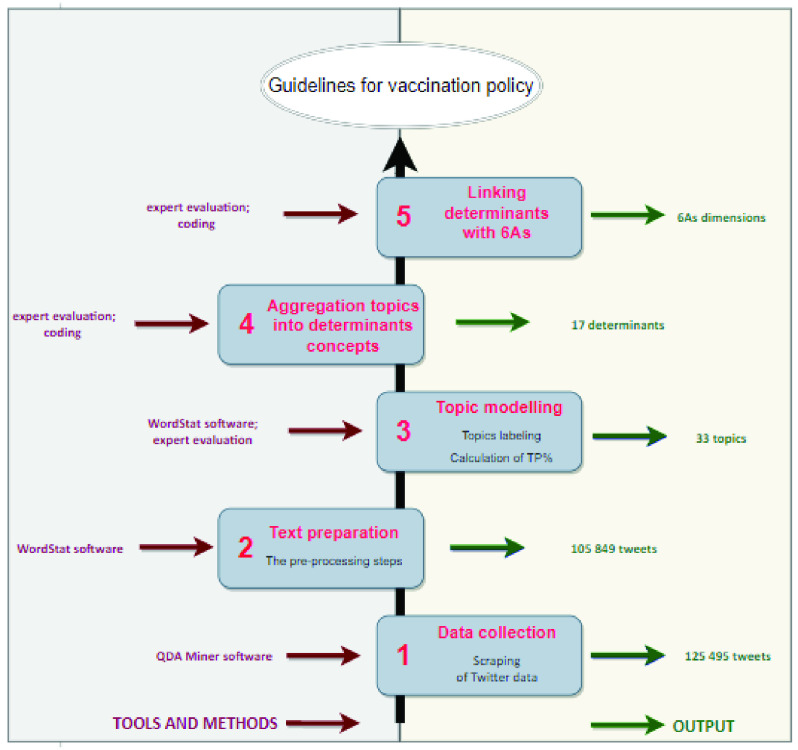
The bottom-up approach: from Twitter data to 6As dimensions of vaccine uptake.

The methodology developed for this study is presented in Figure 1. The activities performed, and the methods and software used at each stage of the bottom-up approach are discussed in more detail below.

#### 1) Data Collection

Involves sourcing relevant data according to a chosen set of keywords and a defined time period. For this study they have already been determined in *Section* $A$. Data collection was conducted using the commercial software QDA Miner, which is part of the ProSuite software [45]. The ProSuite program provides advanced tools for a thorough analysis of data and consists of the following modules: (i) QDA Miner for qualitative data analysis, (ii) WordStat intended for content analysis and text mining, (iii) SimStat designed for statistical analysis. It also offers the option of scraping tweets. In other words, data extraction from Twitter was automated with QDA Miner. In total, 125 495 tweets were collected in this phase. The following information for each tweet was downloaded: (i) tweet full text, (ii) the numbers of favorites and retweets, (iii) user geolocation; (iv) user description/self-created profile, (v) tweet date and hour. In order to check whether the data are balanced, we divided all tweets into subsets (covering a period of 7 days) to identify tweets in the material in terms of a place and date of publication. The content in each subset was then compared to see if the data were evenly distributed. This experiment proved that the data was well balanced. It should be stressed that the research material collected at this stage is represented by unorganized data, with colloquial language, slang, abbreviations or extensions, etc. Thus, the subsequent stage of preparing the data is needed.

#### 2) Text Preparation

Consists of the following tasks: (a) parsing, which means analyzing data and breaking them down into smaller blocks, which separately can be easily interpreted and managed; and (b) preprocessing, also called text cleaning of data, which includes the following jobs: (i) tokenization, where the words are transformed from the text into structured sets of elements called tokens; (ii) compiling a stop word list, where the words which have low informative value or are semantically insignificant (e.g. and, a, or, the) are eliminated; and (iii) stemming, where the words are reduced to their basic form, i.e. word stems are identified. At this phase, we used the WordStat software. We also detected the language of the tweets and retained only tweets in Polish resulting in a dataset with 105 849 tweet documents for further analysis.

#### 3) Topic Modeling

is a method for finding a group of words (i.e topic) from a collection of documents. This is a way to achieve recurring patterns of words in textual data. There are many techniques possible to obtain topic models (e.g. the Latent Dirichlet Allocation, LDA). However, ours was based on an algorithm implemented in the WordStat software. Unsupervised learning was chosen because it is commonly used and allows us to conduct exploratory analyses of large text data in social science research [47]. As a result of topic modeling with the usage of the WordStat software, 33 topics, described by top-weighted keywords, were obtained. Next, an iterative process of topic labeling was performed: (i) topics were labeled to create the first version of labels based on the keywords with the greatest weight, (ii) names of labels were polished through in-depth reading of the most representative topic tweets, and (iii) the final set of topic labels was created. Similar to [47], [49] and [52], our thematic approach relied on human interpretation. Thus, this approach could be significantly influenced by personal understanding of the topics and a variety of biases. The results of this stage are part of the supplementary material: Table B. Next, the proportions of occurring topics were calculated as a percentage (TP, %).

#### 4) Aggregation of Topics into Determinant Concepts

As a result of an in-depth analysis of textual material, by aggregating topics we created 17 determinants from 33 topics representing some kind of problem. It was assumed that each problem/topic could be linked by several determinants. So-called card sorting [53], which means that each topic written on an individual card was assigned to a logically coherent group, was used for creating a determinant. Then the obtained data were entered into the table. The results were presented in the supplementary material: Table C and Table D. Two determinants outside the 5As framework were revealed at this stage. These were referred to as *Protection* and *Insurance*. A similar type of topic classification, not into determinants but overriding themes, was done in the works [47], [52] and [49]. There are many approaches for extracting knowledge from a short text (tweets). A comparison of selected research approaches can be traced in Table 3.

**TABLE 3:** Comparison of Research Approaches

No	Authors	Search query	Method of data collection	Software used	Aim of data analysis	A qualitative approach to develop themes further
1	[47]	Using a list of 25 hashtags as search terms to fetch tweets (e.g. #coronavirus, #2019nCoV, #COVID19, #coronaoutbreak, #quarantine, #StayHome, #SARsCov2, etc.)	Twitter’s open application programming interface (API)	Python and the NRC Emotion Lexicon	13 topics were identified by topic modeling, and sentiment analysis (SA) was performed.	(1) Becoming familiar with the keyword data, (2) generating initial codes, (3) searching for themes, (4) reviewing potential themes, (5) defining themes, and (6) reporting. The thematic approach relied on human interpretation. Coding in NVivo.
2	[50]	Explicit Covid-19 keywords such as “coronavirus”, and keywords such as “school” and “cancelled” in order to include tweets about a wider array of topics impacted by the pandemic.	Twitter streaming API	ANOVA, VADER software	Clustering & subclustering and sentiment analysis Clustering techniques organized tweet data into 15 high-level themes and 15 specific topics within each theme.	(1) Tweets were embedded with Universal Sentence Encoder, (2) a single label was computed using the eight words with the highest overall frequencies, (3) then manual annotations were provided of the prominent themes that arose, by inspecting small samples of tweets within each cluster. To augment human interpretations of each cluster and subcluster, the authors generated summaries using DistilBART, which aims to generate concise summaries without relying on extractive summarization strategies. The BART-based decoder.
3	[52]	A combination of relevant keywords and hashtags: (#covid OR covid OR #covid19 OR covid19) AND (#vaccine OR vaccine OR #vacine OR vaccine OR vaccinate OR immunization OR immune OR vax).	Not mentioned	Python	Behavioral intentions regarding Covid-19 vaccines were mapped to constructs (capability, opportunity, motivation) in the adapted COM-B model.	(1) The coding schema was developed iteratively based on the definitions of constructs in the adapted COM-B model, (2) two reviewers independently coded 1000 tweets in each round, (3) after completing one round of coding, the two reviewers met with a third reviewer to discuss disagreements and update the coding schema until a consensus was reached. The thematic approach relied on human interpretation. The coding tool was not mentioned.
4	[49]	13 keywords: COVID19, CoronavirusPandemic, COVID-19, 2019nCoV, CoronaOutbreak, coronavirus, WuhanVirus, covid19, coronaviruspandemic, covid-19, 2019ncov, coronaoutbreak, and wuhanvirus.	Tweets were collected by the Panacea Lab	R, RStudio Version 1.4.1103 and the National Research Council of Canada Emotion Lexicon	16 topics were identified by topic modelling and SA was performed.	(1) The textmineR package topic label function was used to generate initial labeling for the topics, (2) the authors labeled topics by reading representative tweets for each topic, (3) through discussions, they further grouped the topics into 5 overarching themes. The thematic approach relied on human interpretation. The coding tool was not mentioned.
5	Present work	Keywords: “covid-19” OR “vaccination” OR “vaccine” OR “covid” OR “coronavirus” OR “SARS-CoV-2” OR “Johnson & Johnson” OR “Moderna” OR “Oxford / AstraZeneca” OR “Pfizer / BioNTech”.	QDA Miner software	ProSuite software	33 topics were identified by topic modeling and then the topics were mapped to determinants.	(1) Topics were labeled to create the first version of labels based on the keywords with the greatest weight, (2) the names of labels were polished through in-depth reading of the most representative topic tweets, and (3) a final set of topic labels was created. The thematic approach relied on human interpretation. Coding in Excel.

Note: For more information on the details of each study (e.g. the goal, findings, etc.), see Table 1.

#### 5) Linking Determinants With 6As

Having applied the method used in the previous stage, 17 determinants were assigned to suitable dimensions of the 5As model. This analysis resulted in discovering an additional dimension, which was labeled *Assurance*. Thus, the research extended the model to 6As. The main topics (including the determinants of vaccine uptake emerging from the tweet topics) along with examples of comments were included in Table 5 in Appendix.

**TABLE 4:** The Contributing Factors of Immunization Uptake Identified Under Each of the 6As Dimensions

Name of As group	Determinants
1. Access	1.1. Convenience access
	1.2. Clear procedures and regulations
	1.3. Location of vaccination
	1.4. Help and facilities from the government
2. Affordability	2.1. Price of additional services
	2.2. Time costs
3. Awareness	3.1. Availability of actual information
	3.2. Knowledge about vaccines
	3.3. Consideration of the vaccination and its side effects
	3.4. Knowledge of the vaccination schedule
4. Acceptance	4.1. Perceived vaccine safety
	4.2. Perceived vaccine efficacy
5. Activation	5.1. Prompts and reminders
	5.2. Workplace policies
	5.3. Incentives
6. Assurance	6.1. Protection
	6.2. Insurance

**TABLE 5:** Determinants of Vaccine Uptake Emerging From Tweet Topics Along With Examples of Comments Highly Associated With the Topics (Original Spelling)

Name of As group	Determinants	TP(%)	Topics(problems)	Sample comments in Polish	Sample comments in English
1. Access	1.1.Convenienceaccess	6,1%	Problems with scheduling vaccinations and long queues	„Przyj echalem na szczepienie, ale szkoda ze szczepionki nie przyj echaty	”1 came for a vaccination, but it is a pity that the vaccines did not come
				„@szczepimysie @Dawid95001263 Az Moderna tez sq jakieá problemy z dostaw^? Tacie kolejny raz przesun?li termin 2 dawki, zaraz zbliza si? data graniczna jej podania.W punkcie mówi^, ze jest straszny balagan z dostawami i Modemy na 2.dawk? nie dostaj^ i musz^ przedkladac. @szczepimysie znacie Panstwo problem?”	“@szczepimysie @ Dawid95001263 And there are also some problems with the delivery with Modema? Dads have postponed the second dose once again, the cut-off date of its administration is approaching. At this point, they say that there is a terrible mess with supplies and Modemy for the 2nd dose are not getting and they have to submit. @szczepimysie Do you know the problem?”
			Delays in vaccine deliveries	„@belchatowiec Dzi?ki! Póki co, mam nadziej?, ze to szczepienie wgl. dojdzie do skutku, bo wczoraj do mnie dzwonili, zebym nie przychodzila na umówionq godzin?, tylko pózniej, bo nie wiedzq, kiedy dostawa dojedzie’	”@belchatowiec Thanks! For now, hopefully this vaccination will happen, because yesterday they called me so that I would not come at the agreed time, but later, because they do not know when the delivery will arrive”
				„Michal Dworczyk: #AstraZeneca kolejny raz opóznia dostawy. Firma poinformowala, ze 800 tys. dawek szczepionki nie zostanie dostarczone. Zdecydowalismy, ze w czerwcu b?dziemy klasc nacisk na szczepienia innymi preparatami”	“Michal Dworczyk: #AstraZeneca is delaying deliveries once again. The company announced that 800,000 vaccine doses will not be delivered. We have decided that in June we will emphasize vaccinations with other preparations”
	1.2. Clear procedures and regulations	9,1 %	Inclusion of immobile and non-digital groups	“Jak starsze osoby majq si? zaszczepic, skoro nawet jak ktos pomoze im si? zarejestrowac, to potem termin jest kilkakrotnie zmieniany i powiadomienie przychodzi SMSem, który nie kazda starsza osoba potrafi odczytac? Wtedy termin przepada. W ostatnich dniach jest jakaá plaga zmieniania terminu. Sam dostalem przez dwa dni juz trzy SMSy ze zmian^ terminu.”	“How are older people supposed to get vaccinated, if even if someone helps them register, then the deadline is changed several times and the notification comes via SMS, which not every elderly person can read? Then the deadline is lost. In the last days, there is a plague of changing the date. For two days I have already received three SMSes with the rescheduling.”
2. Affordability			Consideration of people from the risk group	“Dlaczego bijemy kolejne rekordy smiertelnosci na Covid-19? Min. z powodu ghipiej i bezdusznej polityki szczepieñ. M^dre kraje szczepiq w pierwszej kolejnosci najbardziej narazonych najstarszych. U nas szczepieni s^ o wiele liczniej mniej narazeni mlodzi, kosztem najstarszych. https://t.co/4fqdb8YImL”	“Why are we breaking new records for Covid-19 mortality? For example, because of a stupid and heartless vaccination policy. Wise countries vaccinate the most vulnerable first. In our country, the young are vaccinated much more often, at the expense of the oldest, https://t.co/4fqdb8YImL”
			Organization of vaccinations for partially disabled	„Od 25 maja wszystkie osoby z orzeczeniem o umiarkowanym stopniu niepelnosprawnoáci b?dq mogly zaszczepié si? w punktach szczepieñ powszechnych poza kolejkq. W spotkaniu z @michaldworczyk brala udzial równiez Prezes @Koalicja_na https://t.co/sFOnrOhZWF”	“From 25 May, all persons with a certificate of moderate disability will be able to vaccinate at common vaccination centres outside the queue. The President of @Koalicja_na https://t.co/sF0nrOhZWF also participated in the meeting with @michaldworczyk”
	13. Location of vaccination	3,0 %	More vaccination points and closer to villages	“"Mam 46 lat, jestem juz zaszczepiony. Moja mama, lat 80, nie jest. Za pierwszym razem bylo za daleko, bo w miascie oddalonym o 160km. Za drugim razem bylo ok ale….nie dowiezli szczepionek. Za trzecim razem byla chora. Czekamy na czwarty termin."”	“I am 46 years old, I am already vaccinated. My mother, 80, is not. The first time was too far, because in a city 160 km away. The second time was ok, but… they did not deliver vaccines. The third time was sick. We are waiting for the fourth date. ”
	1.4. Help and facilities from thegovernment	9,1%	Mobile home vaccination teams	„RT @zych_jacek: #walbrzych osoby 60+ b?d^ mogly zaszczepic si? we wlasnym domu szczepionka Johnson&Johnson - zadeklarowal prezydent Walbrzycha"	"RT @zychjacek: #walbrzych people 60+ will be able to get vaccinated in their own home with the Johnson & Johnson vaccine - declared the president of Walbrzych"
			Assistance with registration and getting to the vaccination points	“RT @WalbrzychMM: Ruszamy z akcj^ szczepieñ domowych dla walbrzyszan 60+. Jezeli jesteácie Pañstwo tak% osob%. lub znacie osob?. która chce która chce si? zaszczepié w domu. dzwoñcie: 74 664 09 09. Osoby takie b?d4 szczepione jednodawkowym preparatem firmy Johnson & Johnson”	“RT @WalbrzychMM: We are starting a home vaccination campaign for the inhabitants of Walbrzych 60+. If you are such a person, or you know the person, who wants to get vaccinated at home, call: 74 664 09 09. Such persons will be vaccinated with a single-dose preparation of Johnson & Johnson"
			Improvement logistic issues of moving vaccines between vaccination points	“@JacekJaworskiGd @awa2501 @KasiakMarek @michaldworczyk Prawdq jest to co pisaliámy wyzej i powtarzamy to wielokrotnie. Z uwagi na zaburzenia dostaw niektóre terminy prewencyjnie zostaly przesuni?te. Proponowane alokacje zmienialy si? bardzo cz?sto. Dlatego konieczne bylo przesuni?cie, które pozwoli na szczepienie w ramach ChPL”	”@JacekJaworskiGd @ awa2501 @KasiakMarek @michaldworczyk It is true what we wrote above and we repeat it many times. Due to supply disruptions, some preventive deadlines have been postponed. The proposed allocations changed very frequently. Therefore, an offset was needed that would allow vaccination under the ChPL”
	2.1. Price of additional services	6,1%	Assistance with registration and getting to the vaccination points	„Moze starszym trzeba pomóc w szybkim zarejestrowaniu si? na szczepienie? Z jednego numeru tel mozna zapisac trzy osoby, w smartfonie szybko znalezc punkt szczepien najwygodniejszy día seniora. Po prostu, rozejrzec si? wokól i pomóc.”	"Maybe the elderly need help in quickly registering for vaccination? Three people can be registered from one phone number, and a smartphone can quickly find the most convenient vaccination point for a senior. Just take a look around and help.”
			Mobile home vaccination teams	„Samorzqdy b?dq zobowiqzane do nawiqzania kontaktu z seniorami 70 + i rozmow? o szczepieniu przeciw #COVID19. Ch?tnych zaszczepi zespól wyjazdowy - mówi @michaldworczyk @szczepimysie https://t.co/CsYYwQubpJ”	’’Local governments will be required to engage with 70+ seniors and talk about vaccination against # COVID19. The away team will vaccinate those willing - says @michaldworczyk @szczepimysie https://t.co/CsYYwQubpJ”
	2.2. Time costs	3,0%	Lack of clear rules for the vaccination procedure	“@szczepimysie @KasiakMarek Moj maz mial juz dwukrotnie odwolane szczepienie”	”@szczepimysie @KasiakMarek My husband had his vaccination canceled twice”
				“@szczepimysie Witam. W jakim miejscu w Pabianicach ma si? zarejestrowac moja znajoma, która jest alergiczkq i míala kiedys wstrzqs anafilaktyczny. Byla juz zarejestrowana na dzisiaj i poszla na szczepienie ale odmówiono jej szczepienia ze wzgl?du na ryzyko”	“@szczepimysie Hello. Where should my friend who is allergic and had an anaphylactic shock, register in Pabianice? She was already registered for today and went to be immunized but was refused vaccination due to risk”
3. Awareness	3.1.Availability of actual information	12,1%	Low awareness of people of rural and remote areas	“"@michaldworczyk @acosta_re_nata Trzeba zaczqc docierac do matych gmin z akcjq informacyjnq i zmobilizowaó gminne osrodki zdrowia oraz gminne osrodki pomocy spolecznej, zeby dotarli do ludzi 50+. Szczepienie na prowincji chyba nie jest tak bardzo popúlame."	"@michaldworczyk @acosta_re_nata We need to start reaching small municipalities with an information campaign and mobilize municipal health centres and municipal social welfare centres to reach people aged 50+. Vaccination in the provinces is probably not that popular."
				"(…) punkty szczepien szczególnie poza duzymi osrodkami sq trudniej dost?pne i (…) kampania profrekwencyjna pojawia si? tak pózno."	"(…) vaccination points, especially outside large centres, are more difficult to access and (…) the turnout campaign appears so late."
			Volatility and inconsistency of information	“Szczepionkowy zawrót glowy. 17.05 z rana rejestruje mlodego na szczepienie. Najblizszy wolny termin - 24 czerwca. Ok. Popoludniu w telewizorze ludzie z polikliniki mówiq, ze male zainteresowanie i ze sq terminy na juz. Pisz? do nich i okazuje si?, ze terminów jednak nie ma. https://t.co/7YLioLnkA2”	“Vaccine vertigo. At 17.05 in the morning, he registers the young for vaccination. The next available date - June 24. Ok. In the afternoon, on the TV, people from the polyclinic say that there is little interest and that there are deadlines already. I write to them and it turns out that there are no deadlines. https://t.co/7YLioLnkA2"
				„Jak to jest, ze 3 miesiqce temu 'szczepionka' musíala byc przewozona specjalnymi lodówkami w temperaturze -70 i uzyta w ciqgu kilku dni, a dzisiaj moze lezec miesiqc w lodówce. Odst?py w szczepieniu mogq miec kilka tygodni, a t? samq osob? mozna szczepic róznymi wersjami?…???>?”	"How is it that 3 months ago the 'vaccine' had to be transported in special refrigerators at -70 and used within a few days, and today it can be stored in the refrigerator for a month. Vaccination intervals may be several weeks old and the same person can be vaccinated with different versions? … ???>? "
			Lack of transparency of information from the government	“@KlimczewskiPawel @OrdoMedicus @Facebook Czy wie Pan, ze Ministerstwo Zdrowia odmówilo odpowiedzi na pytania lekarzy i naukowców o szczepionk? Covid-19. W przestanej odpowiedzi naczelnik departamentu oswiadczyla, ze postawione pytania nie kwalifikujq si?.”	“@ KlimczewskiPawel @ OrdoMedicus @Facebook Do you know that the Ministry of Health has refused to answer the questions of doctors and scientists about the Covid-19 vaccine. In the reply sent, the head of department stated that the questions posed did not qualify."
			Low quality of shared statistical data posted on the portal	„@PBasiukiewicz Trzeba by w analizie uwzgl?dnió relacje zgonów do liczby szczepien w danym okresie i podziale na przedzialy wiekowe rózniqce si? prawdopodobieñstwem zgonu z przyczyn innych niz szczepienie”	"@PBasiukiewicz The analysis would have to take into account the ratios of deaths to the number of vaccinations in a given period and the division into age groups that differ in the probability of dying for reasons other than vaccination"
	3.2. Knowledge about vaccines	18,2%	Lack of reliable and in-depth knowledge about vaccine biological mechanisms	“@Buster335 Szczepienie ozdrowiencöw ma w ogöle sens? Z tego co mi si? wydaje, to naturalnie wytworzona odpomoic jest lepsza, niz ta wywolana szczepionkq. Byly nawet na ten temat badania, chyba w Izraelu.”	“@ Buster335 Vaccinating convalescents makes sense at all? From what I think naturally produced immunity is better than vaccine induced immunity. There were even studies on it, probably in Israel.”
				„RT @NiezalenyM: Senator Rand Paul oäwiadczyl dzis, ze nie przyjmie szczepionki dopöki nie udowodniq mu, ze szczepienie jest lepsze niz naturalne przechorowanie”	"RT @NiezalenyM: Senator Rand Paul has stated today that he will not be vaccinated until proven to him that vaccination is better than natural disease."
			Fear about longterm effects after vaccination	“RT @maritosal: "CDC bada dziesiqtki zgloszen zapalenia serca u nastolatköw i mlodych doroslych, ktore wyst?pujq cztery dm po drugiej dawce”	"RT @ maritosal:" The CDC is investigating dozens of reports of heart inflammation in adolescents and young adults that occur four days after the second dose "
			Lack of awareness about vaccine adverse reactions	“RT @jzpinski: Przeciez jest oczywiste, ze b?dq zawsze wypadki iz szczepionka jednostkom zaszkodzi, a w skrajnym wypadku moze spowodowac zgon”	"RT @jzpinski: It is obvious, that there will always be accidents that the vaccine will harm individuals, and in extreme cases, it may cause death"
			Detailed knowledge about the effectiveness of vaccines	“RT @SanofiPolska: Jakijestpoziom skutecznoSci szczepionek przeciwko #COVID19 w przypadku indyjskiej, a iaki w przypadku brytyiskiei mutacji?”	“RT @SanofiPolska: What is the effectiveness of the # COVID 19 vaccines for the Indian vaccine, and the British mutation?”
4. Acceptance				“RT @drhalat: Nowe badanie PHE möwi, ze szczepionka AstraZeneca jest skuteczna tylko w 66%. Co si? stalo z „90% osob powyzej 65 roku zycia”	“RT @drhalat: A new PHE study says AstraZeneca is only 66% effective. What Happened to "90% of People Over 65"
				„RT @StarszyLeszek: @KotSzary Brak badan przesiewowych to jedno. Zasadniczym pytaniem jest to, jaki poziom pizeciwcial zapobiega chorobie?”	”RT @StarszyLeszek: @KotSzaryNo screening tests are one thing. The key question is what antibody levels are preventing disease?”
			Lack of relevant information COVID mutations	“RT @KreplewiczRoman: Dla myslqcych inaczej….Nie ma czegos takiego jak szczepionka przeciw koronawirusowa. A dla czego ? BO WIRUS MUTUJE.”	"RT @KreplewiczRoman: For those who think differently …. There is no such thing as a coronavirus vaccine. And for what? BECAUSE THE VIRUS MUTES."
			The need to collect and study data on vaccine side effects	“RT @PBeatap: 59-letnia kobieta ze Szkocji zmarla 48 godzin po zastrzyku AstraZeneca. Otrzymala drugq dawk? eksperymentalnego wektora wirusowego. Dlaczego si? tego nie bada???”	“RT @PBeatap: A 59-year-old female horn Scotland died 48 hours after an AstraZeneca injection. She received a second dose of the experimental viral vector. Why is it not tested ??? "
	3.3.Consideration of thevaccination and its side effects	6,1%	Fear caused by the increased number of deaths after vaccination	“@zdyrmanl @gig2404 Po Pfizerze wczoraj zmarla mama mojej kolezanki. Trzy tyg. po drugiej dawce. Juz po pierwszej wystqpil bol brzucha, kr?goslupa, gorqczka. Zmarla wczoraj rano, corce na r?kach. Wydzielina krwi i plynu z jamy ustnej i z phic. Masakra.”	„@ zdyrmanl @ gig2404 After Pfizer, my friend's mom passed away yesterday. Three weeks after the second dose. Already after the first, there was a pain in the abdomen, spine and fever. She died yesterday morning with her daughter in her arms. Discharge of blood and fluid from the mouth and lungs. Massacre.”
				“@AlicjaJesz Jak nie bylo w sieci NAWET informacji od osob bliskich tym, ktörzy ci?zko przechodzili tzw. covid(nie licz? celebro-covidiotow), tak teraz co i rusz pojawiajq si? w sieci dziesiqtki doniesien o ZGONACH bliskich, znajomych po podaniu eksperymentalnego preparatu!”	“@AlicjaJesz As there was no information on the network EVEN from people close to those who had a hard time going through the so-called covid (I do not count celebro- covidiots), so now dozens of reports about the DEATH of loved ones, friends after administering an experimental preparation appear on the web now and then!”
				„RT @Michali49393358: Zmarla 34- letnia Kalina Mröz, dziennikarka "Gazety Wyborczej". 8 maja pisala o swojej ci?zkiej reakcji na szczepionk?”	"RT @ Michali49393358: 34-year-old Kalina Mroz, journalist of' Gazeta Wyborcza "has passed away. On May 8, she wrote about her severe reaction to the vaccine "
			Concerns of health risks vs usefulness of vaccination	“@OsieckaNguyet @Magorza84783552 @MagdaCDN Moja kuzynka zachorowala na covid po pierwszej dawce, lekaiz zalecil przcsunqc szczepienie drugq dawkq. Takich os6b jest duzo, wiele z nich nie odwoluje szczepienia. Nie wiem czy system ich widzi”	“@OsieckaNguyet @ Magorza84783552 @MagdaCDN My cousin got covid after the first dose, the doctor recommended that the vaccination be delayed with the second dose. There are many such people, many of them do not cancel their vaccination. I don't know if the system sees them"
	3.4. Knowledge of the vaccination	3,0 %	Vaccination schedule issues schedule	“RT @Dawid95001263: Jest 18.05, jesteämy blizej konca niz poczqtku Maja, a wiecie co xd? Dalej nie ma opublikowanego harmonogramu dostaw szczczepien!”	”RT @ Dawid95001263: It's 18.05, we're closer to the end than the beginning of May, and you know what xd? There is still no published vaccination delivery schedule!”
	4.1 Perceived vaccine safety	9,1 %	Mistrust of vaccination safety due to severe effects and mortality after vaccination	“RT @literkal: Szok!- sp6jrzcie Q1 "Ponad 84 tys. osob zakazilo si? koronawirusem juz po szczepieniu wynika z najnowszych danych Ministerstwa”	"RT @ literkal: Shock! - look Q" Over 84,000 people contracted the coronavims after vaccination, according to the latest data from the Ministry.”
				„RT @ChopAntonil: Loteria dla zaszczepionych? Zdziwieni? Przeciez samo szczepienie to loteria. Albo przezyjesz, albo nie”	”RT @ ChopAntonil: Lottery for the vaccinated? Surprised? After all, vaccination itself is a lottery. Either you will live or you will not"
				„RT @Spychacz2: Brytyjka zmarla w wyniku „incydentu zwiqzanego z krzepni^ciem krwi” po wstrzykni^ciu AstraZeneca!! Thank you very much	“RT @ Spychacz2: British girl died from "blood clotting incident" after injecting AstraZeneca !! Thank you very much Liz.”
			Fake medical news and disinformation on Covid-19 vaccines	Liz.” “Zwierz?ta umieraj? po zaszczepieniu szczepionk? przeciwko Covidl9… A co dopiero ludzie? Jackowski slynny polski jasnowidz mowi ze w swoich wizjach widzi duzo slepych ludzi!!! https://t.co/UjLFuJlrT7”	“Animals die after being vaccinated with the Covid 19 vaccine … What about humans? Jackowski, the famous Polish clairvoyant, says that he sees a lot of blind people in his visions !!! https://t.co/UjLFuJlrT7”
				„RT @maritosal: Fizyk i kardiolog nukleamy dr. #RichardFleming: "Covid- 19 i szczepienia Covid to sztuczna bran biologiczna”	”RT @ maritosal: Nuclear physicist and cardiologist Dr. #RichardFleming: "Covid-19 and Covid Vaccinations are an artificial biological weapon”
			Fearing about long-term effects after vaccination	“@MichaZienkiewil @PBasiukiewicz Tego nikt nie wie jaki, i czy w ogole, wplyw moze miec szczepionka na ludzki organizm w dhigiej perspektywie. Szczepieni s^ testerami.”	MichaZienkiewil @PBasiukiewicz Nobody knows what, or if at all, the impact of a vaccine on the human body in the long term. The vaccinated are testers.”
				“Zarowno dfugofalowe skutki po szczepieniu jak i po Covid s? nieznane”	"Both the long-term effects after vaccination and after Covid are unknown."
	4.2. Perceived vaccine efficacy	6,1%	Lack of trust in the vaccine due to the short testing period	“@feirpatak @ann56502906 @BettyElaWhite Moim argumentem jest ofrwiadczenie w grudniu ub.roku Europejskiej Agencji Lek6w,ze szczepionka Pfizer zostala dopuszczona do stosowania WARUNKOWO poniewaz badania trwaj? i zakoncz? si? w 2023 r”	” @feirpatak @ ann56502906 @BettyElaWhite My argument is the statement in December last year by the European Medicines Agency that the Pfizer vaccine was CONDITIONALLY approved because the research is ongoing and will end in 2023”
			Low perception of the population against the effectiveness of vaccines	“@PBasiukiewicz Panie Doktorze dlaczego nie analizuje si? systemu immunologicznego tych, kt6rzy "giadko" przechodz? covid do tych ktdrzy sobie nie radz?? Takie porownanie daioby odpowiedz co organizm potr/ebuje, jakich witamin, substancji, aby bye odpomym. Tak powinna wyglada walka z wimsem”	“@PBasiukiewicz Doctor why is the immune system not analyzed of those who pass covid "smoothly" to those who cannot cope? Such a comparison would answer what the body needs, what vitamins, substances to be resistant to. This is how the fight against the virus should look like”
5. Activation	5.1 Prompts and reminders	3,0 %	The need for direct (or telephone) contact with seniors for vaccination	“@marko_karolina @wszczeklik @FinancialTimes Jesli chodzi o 60+ to bezposredni kontakt z POZ i zaproszenie na szczepienie rozwi?zatby problem. Jesli chodzi o 60- to sprawa jest raezej przegrana.”	“@marko_karolina @wszczeklik @FinancialTimes When it comes to 60+, direct contact with POZ and an invitation to vaccinate would solve the problem. As for the 60's, the case is rather lost.”
			Lack ofcontextualizationadvertising	“Szczepionka to nie jogurt, a troch? tak prdbuje si? to reklamowad?? #szczepimvsi?”	“The vaccine isn’t yogurt, but that’s a bit how it’s advertised?? #szczepimysi?”
	5.2 Workplace policies	3,0 %	The idea of compulsory vaccination	“Samorz?dowcy apeluj? o obowi^zkowe szczepienia. List do rz?du https://t.co/3 5nrAPetGd”	’’Local government officials are calling for compulsory vaccinations. Letter to the government https://t.co/3 5nrAPetGd”
	5.3. Incentives	6,1 %	Lottery and Covid certificates	“Rz?d kombinuje i przedstawia rozne pomysiy, by zach?ci6 Polak6w do szczepienia si? przeciw Covid-19.Jednym z nich ma byi loteria dla os6b zaszczepionych. Loteria przygotowywana jest wraz z Totalizatorem Sportowym.https://t.co/LN94idUg4w”	“The government is combining and presenting various ideas to encourage Poles to vaccinate against Covid-19. One of them is to be a lottery for vaccinated people. The lottery is being prepared together with Totalizator Sportowy.https://t.co/LN94idUg4w”
			Development of incentive measures for vaccination	„Bawi mnie fakt, ze Uber zach?ca do szczepiefi i oferuje mi kod znizkowy na dojazd do punktu, bo jakjechaiam z chlopemna jego szczepienie kierowca Ubera zamilkl kiedy usiyszai gdzie jedziemy, chociaz byl bardzo rozmowny jeszcze chwil? wczesniej xD”	"I am amused by the fact that Uber encourages vaccinations and offers me a discount code to get to the point, because when I was going with the peasant for his vaccination, the Uber driver fell silent when he heard where we were going, although he was very talkative a moment earlier xD"
6. Assurance	6.1. Protection	3,0 %	Discrimination against people, who are not vaccinated	“@JeremiW_Gdansk @MarcinRola89 ubezpieczenie na zycie podobno nie obejmuje smierci na covid.”	”@JeremiW_Gdansk @ MarcinRola89 life insurance reportedly does not cover death from covid.”
				„Chory na gruzlic? nie moze bye kucharzem, wi?c niezaszczepiony przeciw Covid powinien równiez mieé ograniczony dost?p do pewnych funkcji. No brawo, geniusze w rz^dzie, geniusze wsz?dzie https://t.co/H53Sw6KPue”	”A person with tuberculosis cannot be a cook, so an unvaccinated person against Covid should also have limited access to certain functions. Well done, geniuses in government, geniuses everywhere https://t.co/H53 Sw6KPue”
				„RT @KonradBerkowicz: Doradca premierà, który proponuje godzin? policyjn^ i zakaz przemieszczania si? dia niezaszczepionych”	"RT @KonradBerkowicz: Advisor to the prime minister who proposes a curfew and a travel ban for the unvaccinated"
	6.2. Insurance	6.1 %	Lack of insurance for the severe vaccine adverse reactions	“@MariaLe85219860 @iga_swiatek Szczepionka „odpowiednio siina”? mniej siine i bardziej siine? Moze, jeszcze powiesz, ze szczepionka chroni przed zachorowaniem, przeszla pelen termin testów oraz, ze ubezpieczalnie wyplac§ odszkodowania? https://t.co/KzlTygjzlV”	“@ MariaLe85219860 @iga_swiatek Vaccine" strong enough "? Are they less strong and more strong? Maybe you will also say that the vaccine protects you from getting sick, has passed the full test date and that the insurance companies will pay you compensation? https://t.co/KzlTygizlV"
				„RT @Laurunia3: W razie smierci twoja rodzina nie dostanie odszkodowania - Szczepienia na Covid-19 nie obj?te ubezpieczeniami”	”RT @ Laurunia3: In the event of death, your family will not be compensated - Vaccinations against Covid-19 not covered by insurance”
				„Co z Funduszem Kompensacyjnych dla tych co mają powikłania poszczepienne czyli NOPy? co nie ma bo wychodzi że za duzo trzeba by wylożyć, bo tyle tego będzie w przyszłości?”	“What about the Compensation Fund for those who have vaccine complications, i.e. NOPs? what is not because it comes out that too much would have to he put into place, because so much will be in the future?”
			The need for preliminary medical tests before vaccination	“@Tomasz_Obremski Marna miala choroby: nadcisnienie, chorob? wiencow^ NYHA, przebyty bezobjawowo zawal, uchylkowatosc jelita grubego. Zaden z lekarzy nie odradzal i nie widzial przeciwwskazan do szczepienia. Zach?caj^cy do szczepien maj^ krew na r?kach.”	“@Tomasz_Obremski My mother had diseases: hypertension, NYHA coronary disease, a history of asymptomatic infarction, colon diverticulosis. None of the doctors advised against and saw any contraindications to vaccination. They have blood on their hands to encourage vaccinations."

Note: Topics marked in red are characterized by negative sentiment.

By following the steps presented in Fig. 1, it is possible to access relevant knowledge and discover hot threads raised in social media discussions regarding the Covid-19 vaccination. This, in turn, provides a good basis for designing governmental guidelines for improving vaccination policies and increasing their effectiveness.

## IV. Results and Discussion

A set of 33 topics was extracted from the large text dataset representing tweets on the topic of the Covid-19 vaccination. In the next phase of the study, a total of 17 determinants influencing vaccine uptake were identified. They are included in Table 4.

**FIGURE 2.**
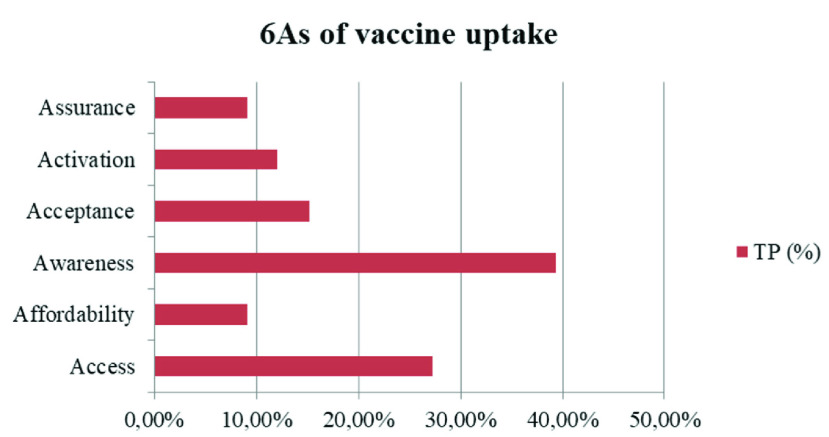
6As dimensions containing the main groups of determinants of Covid-19 vaccine uptake.

Moreover, the list of topics, extended by sample comments providing evidence for each identified factor, is presented in Table 5 (in Appendix). The calculation of topic proportions (TP%) made it possible to calculate the share of each As dimension (Fig. 2).

**FIGURE 3.**
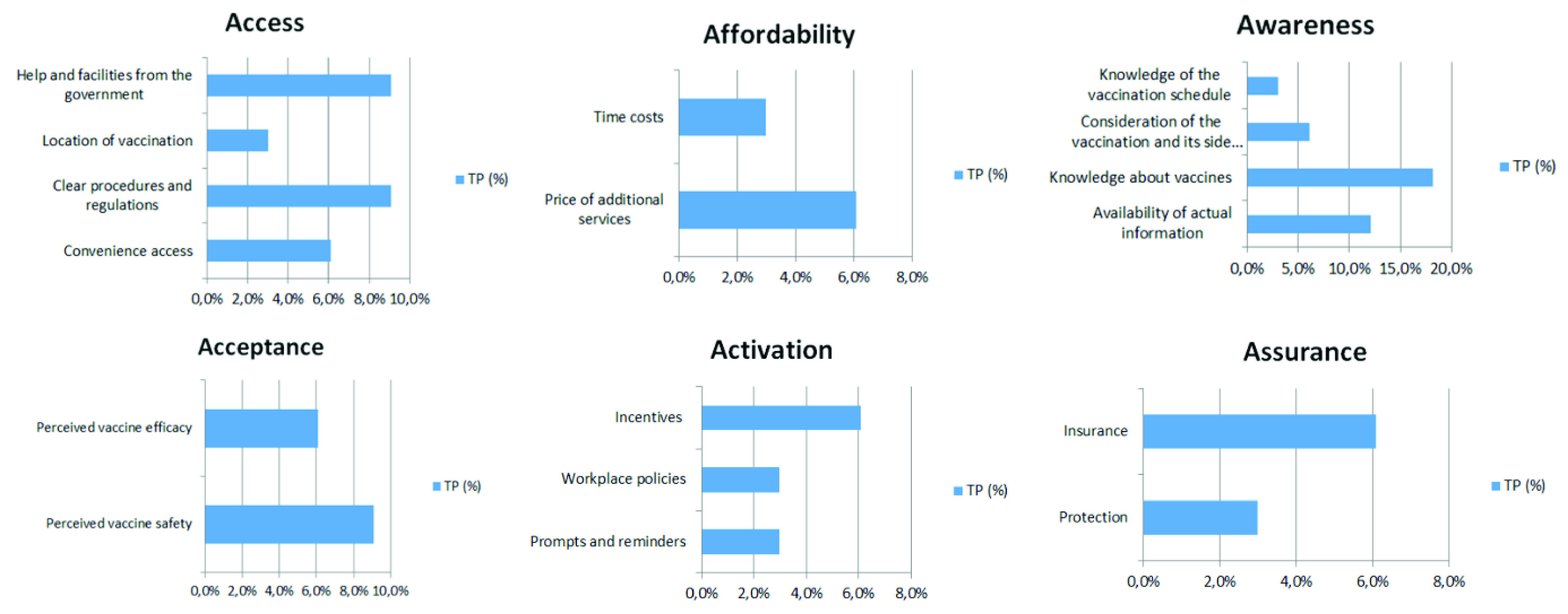
Share of the individual factors identified under each of the 6As dimensions.

The results of this study show that Covid-19 vaccine uptake mostly depends on the dimensions defined as *Awareness* (39.4 %) and *Access* (27.3 %) to the vaccine. *Awareness* covers the availability of a wide range of actual and detailed information regarding vaccines in the population, such as immunization schedules, vaccine side effects, safety and efficacy. Whereas *Access* is linked to the organization of the national vaccination strategy in terms of the following factors: problems with scheduling vaccinations and long queues, delays in vaccine deliveries, poor organization of vaccinations, too few vaccination points, and localization problems, e.g. too far from home. These findings are consistent with the study of [20], who tested Covid-19 vaccine hesitancy in a representative working-age population in France. Their survey experiment showed that detailed knowledge regarding new vaccine characteristics and the national vaccination strategy determine Covid-19 vaccine uptake. The percentage share of all factors identified under each of the 6As dimensions is presented in Fig. 3.

The following subsections summarize the evidence identified for each dimension of the 6As framework.

### A. Access Factors Associated With Vaccine Uptake

According to the WHO’s guidelines, a COVID-19 vaccine allocation strategy should ensure that vaccines are free at the medical point of service, are allocated transparently, and with a participatory prioritization process. Due to the this, vaccines in EU countries are free of charge, so a determinant related to the price was not included in the *Access* group. However, the role of access on vaccine uptake was reflected in obstacles concerning scheduling vaccinations, long queues, and delays in vaccine deliveries. These problems, highlighted in the Twitter discussions, related to the improper organization of many steps in the immunization process, are major barriers to convenient access. Thus, they need urgent improvement and reinforcement.

Another group constitutes unclear procedures and regulations. Many problems were reported in the discussions, such as inconsideration of people from risk groups, exclusion of immobile and non-digital groups, and poor organization of vaccinations for the partially disabled, all of which significantly hinder access to vaccination. The location of vaccination points also had an impact on uptake. Prior studies showed that the organization of vaccinations with convenient access, e.g. in a workplace [22] or at a school [23], results in increased vaccine uptake.

The inclusion of help and facilities from the government is also an important determinant of convenient access to immunization. The analysis of the tweets revealed that, especially in the context of elderly people, there is a lack of assistance with registration and reaching the vaccination points. Mobile home vaccination teams would be a good solution.

### B. Affordability Factors Associated With Vaccine Uptake

The affordability factors identified in the present study consist of two main groups of determinants. First, is the *price of additional services*, which concerns a payment, e.g. assistance with registration and reaching the vaccination points. This is especially true for elderly or disabled people who need the support of third parties to undergo the vaccination procedure. Not everyone can count on free support from their family members. This follows indirectly from the study [40], which found that seniors who lived alone had a lower likelihood of having received the vaccine than those who lived with others. Some have to pay for the help of an assistant in this process.

The second determinant is *time cost*, which is influenced by the lack of clear rules for the vaccination procedure. Twitter comments identify time losses resulting from unclear laws and regulations. An example of such a tweet is: “@Szczepimysie Hello. Where should my friend who is allergic and had an anaphylactic shock, register in Pabianice? She was already registered for today and went to be immunized but was refused vaccination due to risk”.^4^ Prior studies revealed that time cost was a significant predictor of MMR (measles, mumps and rubella) non-vaccination in university students [24], and was a declared disincentive to receive vaccinations in 22% of health professionals surveyed [25].^4^In Polish: “@szczepimysie Witam. W jakim miejscu w Pabianicach ma sie zarejestrować moja znajoma, która jest alergiczka i miała kiedyś wstrzas anafilaktyczny. Była już zarejestrowana na dzisiaj i poszła na szczepienie ale odmówiono jej szczepienia ze wzgledu na ryzyko”.

### C. Awareness Factors Associated With Vaccine Uptake

As mentioned earlier, the determinant group belonging to the *Awareness* dimension covers the largest range (39.4%) in the entire As framework. It groups several threads covered in tweets, constituting ’hot’ topics. Four determinants are included in *Awareness*. First, for increased vaccine uptake, people value the *availability of actual information*. A study of tweets revealed that the continuous volatility and inconsistency of information, the low quality of shared statistical data posted on the administration portal, as well as the lack of transparency of information from the government are factors that need improvement to increase vaccination coverage. The research of [42] stated that respondents reporting higher levels of trust in information from government sources were more likely to be vaccinated.

Second, *detailed knowledge about vaccines* plays a crucial role. This is in line with the work of [26], who found that more knowledge regarding vaccines improved uptake among health professionals. Moreover, according to the study of [25], people who were given more information concerning personal benefits and risks were more likely to be vaccinated.

Third, another diagnosed determinant is *consideration of the vaccination and its side effects*. This determinant was also identified in the research of [1] and [27]. The main topics on Twitter concerned fear caused by the increased number of deaths after vaccination, and captured the health risks vs. the usefulness of vaccination. Our findings are similar to the study of [22], who proved that the main reasons given for not receiving the vaccine were the belief that it had significant side effects, and concerns about its effectiveness.

Finally, the last factor was the *awareness of the vaccination schedule*. Lack of knowledge in this area is an obvious factor contributing to non-vaccination. Thus, thorough information campaigns are necessary so that people do not have to undertake a long search for where to go and at what times to get vaccinated. This is in line with [32], who pointed to an important factor being campaigns, which support people in gaining proper information and help build effective community engagement, and local vaccine acceptability and confidence.

### D. Acceptance Factors Associated With Vaccine Uptake

In the present study, the *Acceptance* dimension, comprising 15.2%, consists of two determinants (i) *perceived vaccine safety*, and (ii) *perceived vaccine efficacy*. Many studies confirm that safety concerns and vaccine side effects contribute to a decline in vaccine uptake in the population [40], [35], [21], [29], [25], [22], [28]. Similarly, belief in vaccine efficacy was an important factor of vaccine uptake [22], [30], [25], [40].

**FIGURE 4.**
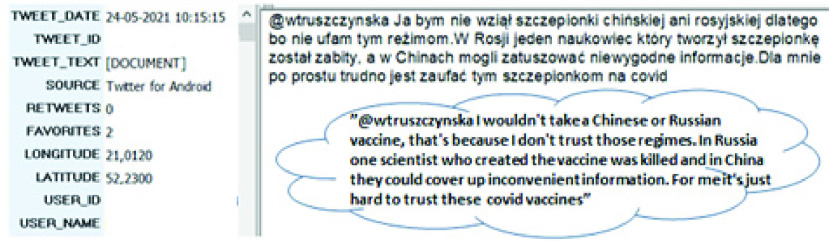
Preview of a single tweet in QDA Miner with the translation (all user-related information has been deleted).

In addition, we found that inconsistent risk messages in terms of the Covid-19 vaccine safety and efficacy from officials, public health experts and individuals, which were expressed in mass media, may contribute to a decrease in the acceptance of vaccination, due to a decline of confidence. This is consistent with the study of [21], who found distrust in vaccine safety to be a crucial determinant of Covid-19 vaccine hesitancy. Twitter users often expressed opinions about vaccine safety and questioned its effectiveness due not only to vaccine novelty, but also other factors (Fig. 4).

There is agreement with many prior studies [2], [25], [26] and [28] that efficacy and safety concerns, including side effects associated with vaccination, can have hugely detrimental effects on the uptake.

### E. Activation Factors Associated With Vaccine Uptake

Activation refers to the actions taken that will make individuals more likely to receive vaccines. Three types of incentive techniques have been identified to stimulate activation: (i) *prompts and reminders*, (ii) *workplace policies*, (iii) *incentives*. The first group included two topics with negative sentiment. The need for direct (or telephone) contact especially with seniors regarding vaccination was pointed out, as this group is constantly overlooked in government programs due to digital exclusion [34], [44]. This result is also consistent with [40], who revealed that receiving a reminder from a doctor (67.7%) was an important influence on accepting a vaccination. According to [22], providing reminders to staff in aged care facilities significantly increased influenza vaccine uptake. Thus, sending reminders about vaccination terms to people is a good idea, and according to [33], for the elderly generation, also in the form of a personal letter. In addition, another theme of negative sentiment was the lack of contextualization advertising, best represented by the tweet: “The vaccine isn’t yogurt, but that’s a bit how it’s advertised??”.^5^ In this area, an important element for improvement is the creation of thoughtful advertising. To support an effective launch of new Covid-19 vaccines, a government needs to understand the community’s concerns, and design such advertising strategies that will neutralize them, and eventually encourage vaccine uptake. Since “one size does not fit all”, the work of [41] recommended avoiding generic messages and instead, considering the different emotional states of the community in tailored vaccine communication efforts.^5^In Polish:”Szczepionka to nie jogurt, a troche tak próbuje sie to reklamować??”

Another determinant, labeled as *Workplace policies,* included the idea of compulsory vaccination, especially in certain professions (e.g. compulsory vaccination for all medical personnel and teachers). The tone of the tweets reflected the split of opinions on mandatory vaccination from acceptance to outright rejection of such a proposal. Examples were shared of forced vaccination by some employers, and the legal implications of this approach were discussed. The study of [38] suggests that obligatory mandates of the Covid-19 vaccination may be ineffective or, worse still, induce a backlash. In turn, the research of [42] reported that 48.1% of respondents would accept their employer’s recommendation to vaccinate. They also claimed an attentive balance is required between educating the public about the necessity for universal vaccine coverage and avoiding any suggestion of coercion.

Finally, the last group of determinants, called *Incentives,* covered such encouragements as lotteries, Covid certificates, and the development of incentive measures for vaccination (e.g. a discount code to get to the vaccination point). When planning vaccination policies, it is worth considering in-depth the strategy for introducing incentives, as the study of [35] found that financial incentives failed to increase vaccination willingness across income levels. Moreover, [36] claimed that payment for vaccination is morally suspect, likely unnecessary, and may be counterproductive. Similarly, [39] argued that financial incentives are likely to discourage vaccination (particularly among those most concerned about adverse effects), and instead, contingent nonfinancial incentives are the desired approach.

### F. Assurance Factors Associated With Vaccine Uptake

A few topics mentioned factors associated with vaccine uptake which were not anticipated by the 5As taxonomy, triggering a sixth dimension, which we labeled *Assurance*. Three main themes emerged in this dimension: (i) discrimination against people who are not vaccinated, (ii) lack of insurance for severe vaccine adverse reactions, (iii) the need for preliminary medical tests before vaccination. The first of these created the *Protection* determinant, which includes comments presenting a wide range of discrimination against unvaccinated people (e.g. a curfew and travel ban for the unvaccinated, etc.). According to the public health principle of least harm to achieve a public health goal, policymakers should implement the option that least impairs individual liberties [43]. The next two topics were labeled *Insurance*. In this group of tweets, there were threads related to the lack of compensation in the case of death related to the Covid-19 vaccination, and insurance in the event of vaccine complications. The necessity of testing people before the vaccination itself was also indicated to diagnose possible contraindications and eliminate post-vaccination complications.

Taking action in the scope described above would certainly increase confidence and contribute to increased vaccine uptake in the population. [37] examined whether compensation can significantly increase Covid-19 vaccine demand. The results showed that, for vaccines, compensation needs to be high enough because low compensation can backfire.

## V. Conclusion

The goal of this study was to determine whether the five dimensions (5As) of *Access*, *Affordability*, *Awareness*, *Acceptance* and *Activation* could correctly cover and organize all the determinants identified from tweets regarding Covid-19 vaccine uptake. This study proved: (i) the existence of a further sixth dimension, labeled *Assurance*; (ii) a preliminary proof-of-concept of the 6As; (iii) the usability and importance of textual data from public discussions in identifying and classifying the different determinants of vaccine uptake. Besides the above-mentioned contributions of this research, another added value to the theory and literature is also the development of the bottom-up methodology used during data analysis.

The empirical part of the present study showed that opinions expressed on social media, i.e. Twitter, constitute a valuable source of data. Knowledge hidden in this information and the discovered relationships should help design immunization campaigns in such a way as to fulfil the suggested needs of citizens and allay their fears as well. Policymakers need to design a well-researched immunization strategy to remove vaccination obstacles, false rumors, and misconceptions regarding the Covid-19 vaccines. Thus, the knowledge of determinants influencing Covid-19 vaccine acceptance can help to create communication strategies that are much needed to strengthen trust in government and health authorities. The study recognized that those interested in vaccination pay the greatest attention to the determinants in the area of *Awareness* and *Acceptance*. For this reason, the promotion of broad and detailed information regarding the vaccines and their side effects, safety and efficacy becomes a key direction in favor of Covid-19 vaccine uptake.

In summary, knowledge about why people avoid the Covid-19 vaccination and which problems could act as obstacles during the immunization process may help government agencies, officials, and other authorities to (i) develop guidance for policies of immunization programs, (ii) create preventative measures against vaccine avoidance, (iii) increase public information campaigns designed to raise confidence in the effectiveness and safety of the vaccine, and finally (iv) design more tailored activities to increase the overall level of vaccine uptake in the population.

However, the present study bears several limitations. First, this research focuses on the discussion via the Twitter platform and includes a short data retrieval period. Data that were collected and reported here are only a snapshot taken at an arbitrarily chosen point in time. These data were scraped in a highly changing environment of social media, with dynamic daily volatility in the perceived threat of the Covid-19 disease and issues of vaccines. Second, the study was narrowed down to only one country. Therefore, a generalization of results is difficult and it can be assumed that other threads may appear on social media discussions depending on the temporal and geographical scope of the study. Third, the study deliberately omitted the performance of a sentiment analysis of tweet data as this was not included in the purpose of the paper. In future, it is worth focusing on a task categorizing tweets for each topic into negative, neutral and positive.

Nevertheless, the 6As taxonomy successfully captured all the determinants of Covid-19 vaccine uptake. Thus, future research may use this taxonomy to structure, classify and compare the significance of each of the 6As in explaining the immunization gap for different vaccines.

In future research, a literature review could also be conducted to reveal current implementation strategies for Covid-19 vaccine promotion and to map them to the 6As framework identified in this study in order to determine gaps in recent research.

## Appendix

See Table 5.
